# Total flavonoids of *Selaginella tamariscina* (P. Beauv.) Spring ameliorates diabetes-induced acute lung injury via activating Nrf2/HO-1

**DOI:** 10.22038/ijbms.2024.79246.17166

**Published:** 2024

**Authors:** Lina Chen, Guosu Xiao, Zhou Yu, Niwen Huang, Yiju Cheng

**Affiliations:** 1 Department of Respiratory and Critical Care Medicine, The Affiliated Hospital of Guizhou Medical University, Guiyang, 550001, Guizhou, China; 2 Department of Clinical Medicine, Guizhou Medical University, Guiyang, 550001, Guizhou, China; 3 Department of Tuberculosis, Guiyang Public Health Clinical Center, Guiyang, 550001, Guizhou, China; 4 Department of Respiratory and Critical Care Medicine, The Fourth People’s Hospital of Guiyang, Guiyang, 550002, Guizhou, China; #T hese authors contributed eqully to this work

**Keywords:** Acute lung injury, Diabetes mellitus, HO-1, Nrf2, Total flavonoids of - Selaginella tamariscina (P.Beauv.) Spring

## Abstract

**Objective(s):**

This investigation explored the mechanism by which the total flavonoids of *Selaginella tamariscina* (P.Beauv.) Spring (TFST) mitigate oxidative stress through the activation of the heme oxygenase-1 (HO-1) signaling pathway mediated by nuclear factor erythroid 2-related factor 2 (Nrf2), thereby ameliorating acute lung injury (ALI) induced by diabetes.

**Materials and Methods:**

Male mice weighing 20–25 grams were divided into four groups: a control group, a diabetic group, a diabetic group treated with TFST, and a diabetic group treated with TFST and ML385. Various biological specimens were collected for analysis, including bronchoalveolar lavage fluid (BALF), blood, and tissue samples. These were subjected to a range of assessments covering hematological and BALF parameters tumor necrosis factor-alpha (TNF-α), interleukin-6 [IL-6]), biochemical markers (malondialdehyde [MDA], superoxide dismutase [SOD], glutathione peroxidase [GSH], Nrf2, and HO-1 levels), along with histopathological evaluations.

**Results:**

Pre-treatment with TFST demonstrated a significant decrease in pulmonary tissue damage, evidenced by decreased wet-to-dry (W/D) lung ratios (*P*<0.001), reduced lung injury scores (*P*<0.0001), and lower levels of TNF-α, IL-6 (*P*<0.0001), as well as oxidative stress markers like MDA (*P*<0.05). Moreover, there was an elevation in the activity of anti-oxidative enzymes, specifically SOD and GSH (*P*<0.05), coupled with an enhanced expression of Nrf2 and HO-1 in the diabetic group (*P*<0.01).

**Conclusion:**

The study findings demonstrate that TFST can suppress oxidative stress by modulating the Nrf2 pathway and up-regulating HO-1 activity, thereby ameliorating diabetes-induced acute lung injury.

## Introduction

Diabetes mellitus (DM) poses a global health challenge, projected to impact 693 million individuals by 2045 (1). DM patients often succumb to complications arising from the disease. DM is characterized as a metabolic disorder with diverse complications (2), including disruptions in glucose metabolism leading to various severe conditions (3). Recent evidence indicates that DM predisposes individuals to ALI (4-6), a condition exacerbated by hyperglycemia-induced inflammation and oxidative stress (7, 8). Despite this, diabetes-related lung injury is frequently overlooked by researchers and healthcare practitioners (9). The aging population’s prolonged exposure to chronic hyperglycemia is anticipated to result in an increase in pulmonary dysfunction due to diabetes (10). ALI, an acute diffuse inflammatory lung injury (11), manifests with increased dyspnea, compromised gas exchange, and loss of lung ventilation tissue (11, 12). Managing ALI is challenging and associated with considerable clinical morbidity and mortality rates. Research has shown that distinct subphenotypes of ALI exhibit varied responses to treatment and are correlated with high mortality rates (13). This heterogeneity may account for the lack of success of promising drugs in phase III trials (14, 15). Identifying patients at a heightened risk of ALI is imperative. Factors such as long-term alcohol consumption, hypoalbuminemia, history of blood transfusions, pulmonary infection sources, and comorbidities like DM (14) regulate the incidence of ALI (16-20). Effective treatment modalities and medications remain inadequate, underscoring the urgent need for thorough research.

Nrf2 is pivotal in bolstering anti-oxidant defense mechanisms and mitigating oxidative damage (21, 22). Under oxidative stress conditions, Nrf2 translocates to the nucleus, regulating the transcription of various anti-oxidative enzymes, including SOD, GSH, and HO-1. This regulation counteracts excess reactive oxygen species (ROS) (23, 24), preserving cellular equilibrium. HO-1, a vital anti-oxidant gene, combats oxidative damage, controls inflammation, and regulates apoptosis (25). Extensive studies on the Nrf2/HO-1 pathway have underscored its significance in combating oxidative stress (26). Hence, therapeutic strategies targeting the activation of these pathways may play a crucial role in attenuating ALI (27).

*Selaginella tamariscina *(P.Beauv.) Spring (TFST) is a folk medicine. The total flavonoids extracted from TFST are also known as TFST. TFST exhibits anti-aging and anti-hyperglycemic properties (28). Research has illustrated their anti-oxidative and lipid-lowering capabilities in diabetic murine models (29-34). Additionally, TFST has shown potential in mitigating inflammation and oxidative stress by modulating MAPK, NF-κB, and Nrf2 signaling pathways (35). However, the utilization of the Nrf2/HO-1 pathway by TFST as a protective mechanism against diabetes-induced acute lung injury in mice remains unexplored. This study aims to elucidate the protective role of TFST against pulmonary injury induced by diabetes and investigate the underlying mechanism of oxidative stress regulation via Nrf2 activation.

## Materials and Methods

***Experimental animals***

Forty male C57BL/6 mice were divided into four groups (n=10 in each group). These mice, weighing 20 to 25 grams and aged eight weeks, were sourced from Guizhou Medical University’s Animal Center. The animals were housed under controlled laboratory conditions, adhering to stringent environmental parameters. They underwent an acclimatization period of one week, during which they had free access to food and water. All experimental procedures were conducted in accordance with ethical guidelines established by the Ethics Committee of Guizhou Medical University’s Faculty of Medicine (Permit Number: 2303434).

***Drugs*** ***&*** ***kits***

TFST was obtained from Hunan Kanglu Biological Technology Co., Ltd (Hunan, China). Streptozotocin (STZ), a nitrosourea derivative, was procured from Sigma Aldrich Co. (St. Louis, MO, USA). Nrf2 polyclonal antibody was acquired from ProteintechTM Biotechnology Co., Ltd (Wuhan, China), while HO-1 rabbit monoclonal antibody was sourced from Beyotime Biotech. Inc. (Shanghai, China). Assay kits for MDA, SOD, and GSH levels were provided by Nanjing Jiancheng Bioengineering Institute (Jiangsu, China).

***Experimental protocol***

Mice were randomly assigned to four groups: control, diabetes, diabetes treated with TFST, and diabetes treated with both TFST and ML385. Except for the control group, the pathology of the lungs in the other three groups needed to be confirmed, and inflammation and inflammatory factors in blood and BALF also increased. There were some criteria for exiting the study: deterioration in the severity of the disease, inappropriateness for continued experimentation owing to adverse reactions, and other reasons preventing the continuation of the study. STZ was administered intraperitoneally to induce diabetes, with blood glucose levels over 16.7 mmol/L indicating diabetic status. The respective treatment groups received TFST at a concentration of 140 mg/kg by gavage for six weeks, with the D+TFST+ML385 group also receiving ML385 intraperitoneally. Blood samples were collected via venipuncture, followed by centrifugation for serum isolation. Tracheal intubation allowed for the collection of BALF, after which biochemical evaluations were conducted on the right pulmonary segment. The left lung segment underwent histological and immunohistochemical analyses.

***Biochemical measurements of blood and*** ***bronchoalveolar lavage fluid***

Quantitative assessments of inflammatory biomarkers in blood and BALF, IL-6, and TNF-α were conducted employing the enzyme-linked immunosorbent assay (ELISA) technique.

***Determination of the W/D ratio of lungs***

The W/D weight ratio of lung tissue served as an index of pulmonary edema. To ascertain the W/D weight ratio, a segment of the right pulmonary tissue was measured for mass, desiccated in a vacuum oven at a temperature of 60 degrees Celsius until reaching a stable mass, and then subjected to a second weighing. 

***HE staining***

Specimens from the left lung were encased within paraffin blocks and sectioned into slices measuring 4–5 micrometers. These slices were then subjected to the standard HE staining protocol. Subsequent to staining, the prepared sections were scrutinized and captured photographically with a light microscope.

***Immunohistochemistry***

The immunohistochemical examination of the left lungs was performed for Nrf2 (dilution: 1:50). The positive expressions were assessed by image J program analysis and were expressed as percentage area.

***Measurement of MDA, SOD, and GSH activities***

The right lung tissue was homogenized in phosphate-buffered saline and assayed for oxidative stress markers. MDA levels were measured colorimetrically, while SOD and GSH activities were also determined colorimetrically.

***Western blot***

Proteomic extraction from the right pulmonary tissues was performed utilizing RIPA lysis buffer sourced from Solarbio (Beijing, China). The protein yield was quantified with a BCA protein assay kit provided by Beyotime (Shanghai, China). Subsequent separation of the proteins occurred through 10% SDS-PAGE supplied by Solarbio (China), followed by their transference to PVDF membranes procured from Millipore (USA). These membranes underwent a blocking phase with 5% skimmed milk at ambient temperature for one hour before an overnight incubation at 4 °C with various primary antibodies (Nrf2 at a dilution of 1:6000; HO-1 at 1:1000). Post-incubation, the membranes were thrice rinsed with TBST and then exposed to a secondary antibody at a dilution of 1:6000 for one hour at ambient temperature. GADPH served as the internal standard. Detection of the target proteins was facilitated by an Enhanced Chemiluminescence (ECL) system, and band intensities were quantitatively assessed using the ImageJ software package.

***Statistical analysis***

Data analysis was conducted using SPSS 20.0 software, and the results were presented as mean ± SD. Differences between groups were analyzed using one-way ANOVA and Tukey’s multiple comparisons test. Non-parametric tests used were the Kruskal-Wallis test and Dunn’s multiple comparisons test. *P*<0.05 was considered to be significant.

## Results

***Effect of TFST on inflammation in diabetic murine pulmonary tissue***

**Figure 1:**
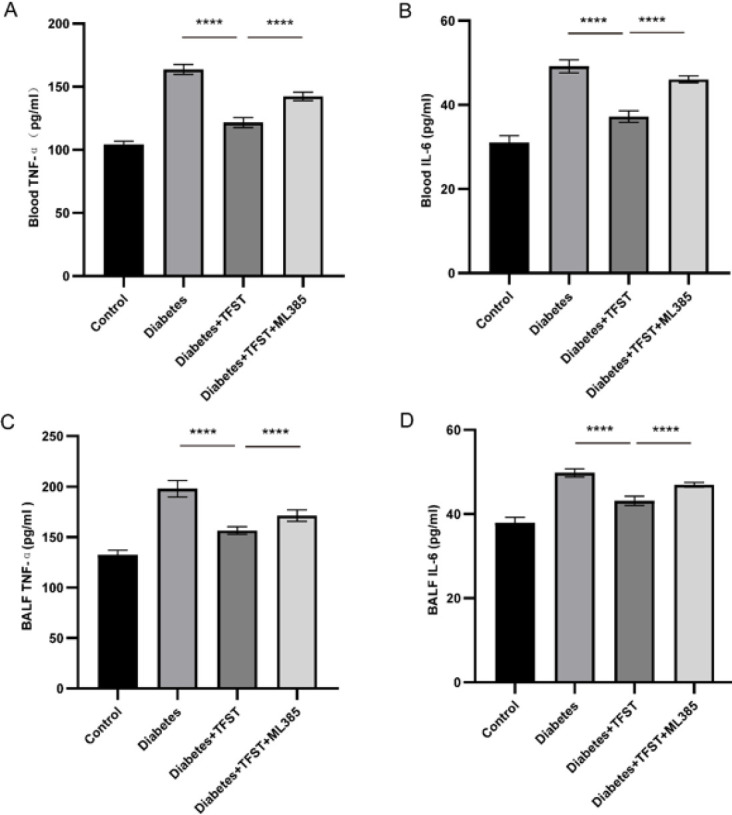
Effects of TFST on TNF-α and IL-6 in blood and BALF

Figure 1 illustrates a significant decrease in the levels of pro-inflammatory cytokines TNF-α and IL-6 in the TFST-treated diabetic group compared to the diabetic control group (*P*<0.0001). Co-administration of ML385 with TFST (D+TFST+ML385 group) led to an increase in TNF-α and IL-6 levels compared to TFST treatment alone (D+TFST) (*P*<0.0001). This trend was consistent in both blood and bronchoalveolar lavage fluid (BALF).

***Anti-oxidant properties of TFST in acute lung injury***

As depicted in Table 1, TFST exhibited anti-oxidative effects in diabetic mouse models. MDA levels were elevated in the diabetic control group, while SOD and GSH levels were significantly reduced compared to the normoglycemic control group. Treatment with TFST in diabetic mice resulted in a significant increase in SOD and GSH concentrations and a decrease in MDA levels (*P*<0.05). Conversely, the D+TFST+ML385 group showed a reduction in SOD and GSH levels and an increase in MDA concentrations compared to the D+TFST group (*P*<0.05).

TFST Ameliorates Diabetes-Induced Acute Lung Injury

**Figure 2:**
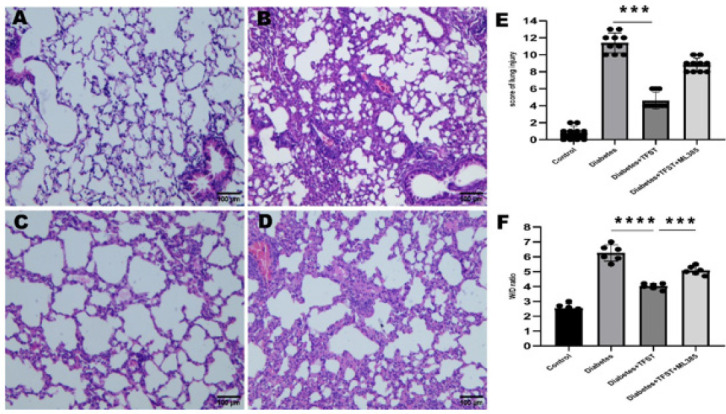
Effects of TFST on histopathological analysis of lung tissue, score of lung injury score, and W/D ratio

Histological analysis presented in Figure 2 showed that the control group (A) and the TFST-treated group (C) maintained normal lung morphology with minimal neutrophil infiltration. In contrast, the diabetic group (B) and the group treated with TFST and ML385 (D) exhibited characteristics of pulmonary damage such as hemorrhage, edema, alveolar wall thickening, and neutrophilic infiltration. TFST treatment alone (C) notably alleviated these pathological features compared to both the diabetic group (B) and the combined therapy group (D+TFST+ML385). These findings were supported by lung injury scores (E) and wet-to-dry weight ratio (F) assessments, consistent with the histological observations.

**Figure 3:**
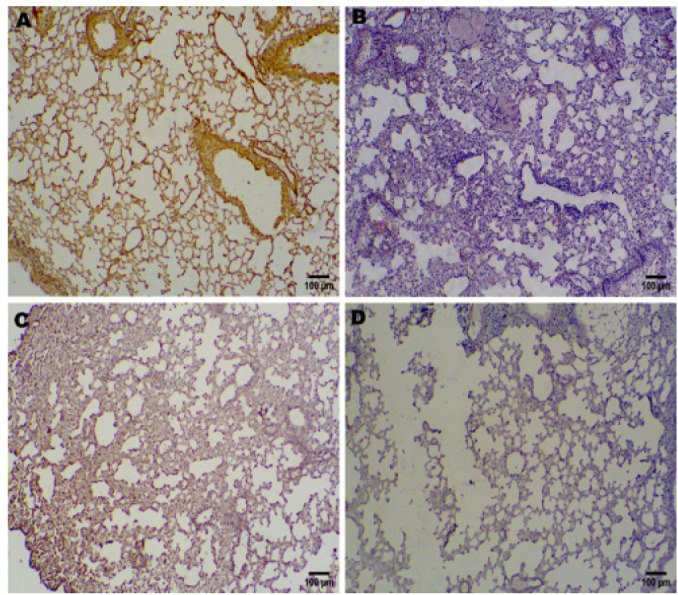
Impacts of TFST on Nrf2 immunohistochemical expression in lung tissue

Effects of TFST on Nrf2 Immunohistochemical Expression in Pulmonary Tissue Figure 3 demonstrates a significant increase in Nrf2 expression in the TFST-treated group (D+TFST) compared to the diabetic group and the group treated with TFST and ML385. Nrf2 expression was notably higher in the TFST-treated group relative to the untreated diabetic mice. Conversely, Nrf2 expression was reduced in the combination treatment group (D+TFST+ML385).

**Figure 4:**
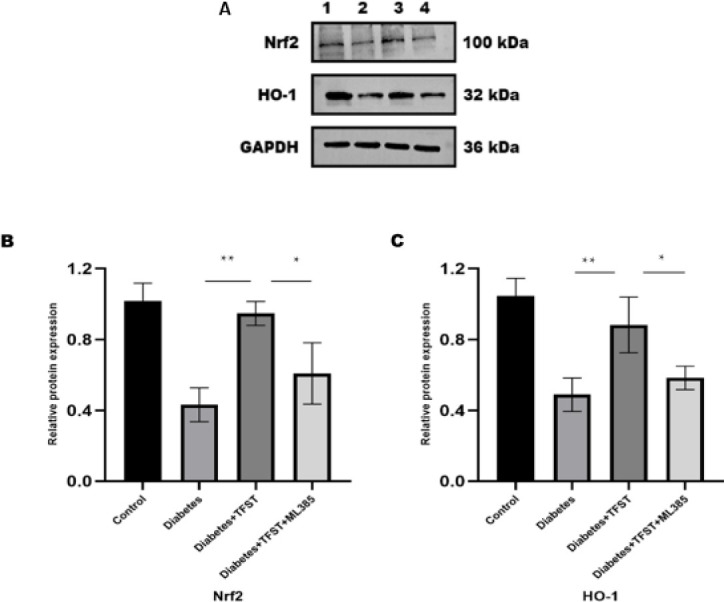
Effects of TFST on Nrf2 and HO-1 protein expression. Both Nrf2 and HO-1 proteins were elevated within the diabetes+TFST group, whereas thatthey were suppressed with ML385

Impact of TFST on Nrf2 and HO-1 Protein Expression in Diabetes-Induced Acute Lung Injury Figure 4, using western blot analysis, revealed higher concentrations of Nrf2 and HO-1 proteins in the TFST-treated group (D+TFST) compared to the untreated diabetic control group. However, the levels of these proteins were decreased in the group receiving both TFST and ML385 (D+TFST+ML385).

## Discussion

Diabetes mellitus, a multifaceted syndrome characterized by persistent hyperglycemia, oxidative stress, and inflammation (36), particularly impacts the lungs, rendering them susceptible to harm (37, 38). This investigation delved into the salutary impact of TFST on acute lung injury induced by diabetes. With TFST intervention, both oxidative stress indicators and histological parameters exhibited enhancement compared to the diabetic cohort, underscoring TFST’s protective role against ALI triggered by diabetes. Moreover, Nrf2 and HO-1 proteins surged within the (D+TFST) group, while these proteins’ levels were dampened in the (D+TFST+ML385) ensemble. Hence, TFST mitigates acute lung injury due to diabetes by activating the Nrf2/HO-1 pathway.

Protracted hyperglycemia catalyzes an excessive generation of ROS and reactive nitrogen species (RNS), capable of inflicting damage on nucleic acids, lipid frameworks, and protein compositions (39-42). Additionally, hyperglycemia-triggered microangiopathy is implicated in the genesis of pulmonary interstitial damage (43). In diabetic individuals, this is often mirrored by thickened basal membranes and escalated inflammatory reactions, disrupting the normal pulmonary tissue architecture (44). Alleviating lung injury depends on maintaining the delicate balance between oxidative stress and anti-oxidant mechanisms (35). Consequently, interventions aimed at oxidative stress pathways have the potential to alleviate lung impairment linked to diabetes. Our study encompassed the establishment of a diabetes-induced acute lung injury model, validated by discernible increments in the W/D ratio and lung injury scores. Histological evaluations unveiled significant alveolar injury, fusion, interstitial thickening, and infiltration of inflammatory cells, indicative of exacerbated pulmonary tissue pathology. Our findings, derived from a diabetic mouse model induced by high-dose STZ injections, align with extant literature on the subject (45).

Hyperglycemia instigates damage to the Nrf2/Kelch-like ECH-related protein 1 (Keap1) pathway (46). Beneath hyperglycemic conditions, Nrf2-regulated anti-oxidant enzyme expression in rat Müller retinal cells wanes (47). Hyperglycemia impedes Nrf2 from binding to Maf proteins in the nucleus (48). The Nrf2/Keap1 signaling axis emerges as a common mechanism implicated in the pancreas of diabetic patients (49). Nrf2 epitomizes a cornerstone in the body’s anti-oxidative defense, orchestrating multiple signaling pathways and integral to an array of anti-oxidant genes and protein repair mechanisms (50). Upon activation, Nrf2 migrates to the nucleus, interacts with anti-oxidant response elements (AREs), and initiates the transcription of defensive proteins like HO-1, curtailing ROS production (51). Consequently, the Nrf2/HO-1 pathway emerges as a pivotal conduit in managing inherent oxidative stress, with its activation demonstrating efficacy in mitigating oxidative harm (52). Evidence points to the modulation of the Nrf2/HO-1 pathway as efficacious in ameliorating acute lung injury (53). Nonetheless, the interplay of the Nrf2/HO-1 pathway activation by TFST in countering diabetes-induced acute lung injury remains unexplored.

TFST finds application in folk medicine for diverse ailments. TFST, derived from traditional herbal origins, boasts a myriad of pharmacological attributes, encompassing anti-inflammatory, anti-airway hyperresponsiveness, anti-oxidant, anti-aging, and hypoglycemic effects (29-34). Investigations validate that TFST effectively impedes the phosphorylation of MAPK and NF-κB, confines NF-κB’s nuclear translocation in macrophages, and simultaneously augments Nrf2 and HO-1 expression (54). TFST’s hypoglycemic effects closely resemble those of metformin hydrochloride sans impact on normal blood glucose levels (30). TFST’s efficacy in enhancing chaotic glucose and lipid metabolism mirrors rosiglitazone effects, indicating promise as a potential diabetic therapy in humans (55). The salutary effects of TFST on glucose and lipid metabolism for T2DM bear semblance to gliclazide’s effects (56). To the best of our knowledge, this report presents the first unveiling of TFST’s effects on diabetic-induced lung injury. Our study underscores TFST’s pivotal role in alleviating diabetes-induced lung injury, corroborated by notable escalations in oxidative strain and inflammatory response bioindicators, including IL-6, TNF-α, MDA, SOD, and GSH. The buoyant impact of TFST further stands affirmed by perceptible enhancements in lung histopathology post-treatment. Western blot analysis elucidated a significant upsurge in Nrf2 and HO-1 protein expression in the diabetic cohort post-TFST treatment relative to both the untreated diabetic group and the TFST with ML385-treated group—an Nrf2 inhibitor. Histological scrutiny of lung tissue sections from the TFST-treated diabetic group (D+TFST) unveiled preservation of normal architecture, minimal interstitial edema, and scant neutrophil infiltration. Conversely, the ensemble receiving both TFST and ML385 (D+TFST+ML385) displayed acute disruption of alveolar epithelial integrity and heightened inflammatory cell accumulation, substantiated by lung tissue microphotographs. Lung injury scores, aligned with the histopathological alterations, signified TFST’s protective efficacy against diabetes-induced acute lung injury, possibly through TFST’s antagonism of oxidative stress via Nrf2/HO-1 pathway regulation.

While clinical studies suggest a protective aspect of diabetes vis-à-vis acute respiratory distress syndrome (ARDS) (20,57,58), some delineate diabetes exacerbating acute lung injury (59,60). Our study aligns with the latter, negating DM’s protective effects. Future studies warrant tailored protocols to scrutinize diabetes’s impact on ALI/ARDS.

Treatment with TFST diminished levels of TNF-α and IL-6, whereas that risedthey were raised in the D+TFST+ML385 group. A) level of TNF-α in blood, B) level of IL-6 in blood, C) level of TNF-α in BALF, and D) level of IL-6 in BALF. Data are expressed as means±SD, n=10.**** *P*<0.0001

TFST: Total flavonoids of *Selaginella tamariscina* (P.Beauv.); BALF: Bronchoalveolar lavage fluid; TNF-α: Tumor necrosis factor-alpha; IL-6

The control group (A) and the TFST-treated group (C) maintained normal lung architecture with minimal or no neutrophil infiltration. In theThe diabetic group (B) and the TFST+ML385-treaded group (D) exhibited histopathological disarrangement, and intense neutrophilic infiltration. E score of lung injury, n=10. F W/D ratio, n=6. dataData are expressed means±SD.*** *P*<0.001, **** *P*<0.0001.

TFST: Total flavonoids of *Selaginella tamariscina* (P.Beauv.); W/D: Wet-to-dry

Nrf2 expression was augmented in the control cohort (A) compared to both the diabetic group (B) and the TFST+ML385-treaded group (D). Nrf2 expression was elevated in the (D+TFST) group (C), whereas it was diminished in the (D+TFST+ML385) group (D), n=6.

TFST: Total flavonoids of *Selaginella tamariscina* (P.Beauv.); Nrf2: Nuclear factor erythroid 2-related factor 2

(A) Representative Westernwestern blots. 1: Control group, 2: Diabetes group, 3: Diabetes+TFST group, 4 Diabetes+TFST+ML385 group (B and C) Changes of density of Nrf2 and HO-1 protein western blot. Data are expressed means±SD, n=6. *P<0.05, * * *P*<0.01.

TFST: Total flavonoids of *Selaginella tamariscina* (P.Beauv.); Nuclear factor erythroid 2-related factor 2

**Table 1:** Anti-oxidant effects of total flavonoids of *Selaginella tamariscina* (P.Beauv.) in lung tissue of diabetes-induced acute lung injury

Group	MDA (nmol/g)	SOD (U/ng)	GSH (nmol/g)
C	76.61±1.75	968.85±10.65	592.22±12.96
D	238.79±4.76	386.68±7.78	187.52±8.14
D+TFST	97.86±1.58^*^	859.56±13.91^*^	448.40±9.53^*^
D+TFST+ML385	140.42±2.48^#^	685.68±16.75^#^	301.68±7.88^#^

Data are expressed means±SD, n=10. *P<0.05 vs. diabetes group; #* P*<0.05 vs. diabetes treated with Total flavonoids of *Selaginella tamariscina* (P.Beauv.)(TFST) group

## Conclusion

TFST can inhibit oxidative stress by regulating the Nrf2 pathway and up-regulating HO-1 activity, thereby improving diabetes-induced acute lung injury. This indicates that TFST shows potential as a therapeutic candidate for mitigating diabetes-induced acute lung injury.
